# 3D Rendering of Cystoscopy Video Footage: A Novel Method Utilizing Neural Radiance Field Processing

**DOI:** 10.7759/cureus.89825

**Published:** 2025-08-11

**Authors:** Jonathan E Katz, Jamie Finegan, Pablo F Beutelspacher, Jingpei Lu, Shan Lin, Michael Yip, Roger L Sur

**Affiliations:** 1 Urology, University of California San Diego, San Diego, USA; 2 Electrical and Computer Engineering, University of California San Diego, San Diego, USA

**Keywords:** 3d rendering, cystoscopy, instant neural graphics primitives, medical imaging, neural radiance fields

## Abstract

Recent developments in neural radiance field (NeRF) processing have leveraged the power of neural networks to quickly reconstruct 3D spaces from 2D images. Our objective was to utilize this technology to 3D render video recordings of diagnostic cystoscopies and test their fidelity. With institutional review board (IRB) approval, we recorded two diagnostic cystoscopies, one with an Ambu single-use flexible cystoscope and the other with a Richard Wolf digital cystoscope. We converted the videos to images and manually curated approximately 100 representative images, which minimized blur and spanned a large segment of the bladder. We then utilized the NVIDIA Instant Neural Graphics Primitives (iNGP), a NeRF algorithm that uses multiresolution hash encoding with a compact neural network for significantly faster convergence, to reconstruct the bladder and render novel, unseen views within seconds. We computed the structural similarity index (SSIM) and peak signal-to-noise ratio (PSNR) to assess the quality and fidelity of the 3D rendering. Both videos were able to be utilized for 3D rendering using iNGP. The rendering derived from the Richard Wolf cystoscopy had a PSNR = 29.8 (min = 27.2, max = 32.6) and SSIM = 0.89. Similarly, the rendering derived from the Ambu cystoscopy had a PSNR = 31.3 (min = 27.1, max = 35.1) and SSIM = 0.90. Independent of cystoscopy equipment, both 3D renderings achieved reasonable fidelity. Major limitations to widespread adoption of this technology include the need for a curator to select representative and high-quality images from the initial cystoscopy video recording and the relatively small segments of bladder successfully rendered. Nonetheless, we feel that with further refinement, this technology can be scaled to create 3D renderings of cystoscopies that will enable evaluation of both completeness and quality of the cystoscopy. Furthermore, this technology would be able to facilitate the comparison of cystoscopies performed in the same patient over time.

## Introduction

Flexible cystoscopy is the gold standard for assessing and diagnosing bladder lesions. Most commonly, this is done as part of the evaluation for patients with either microscopic hematuria or in patients undergoing surveillance for bladder cancer recurrences [1]. However, challenges with this technique persist. Both the urologist's inability to objectively assess the completeness of the procedure and the lack of ability to easily compare bladder lesions over time leave room for technologic innovation to bridge these deficiencies.

During standard flexible cystoscopy, there is no mechanism to objectively assess the completeness of the examination of the bladder. Yet, cystoscopy can only be considered successful if it is being performed completely and accurately. For example, in patients with high-risk non-muscle invasive bladder cancer, where two consecutive cystoscopies are performed within four to six weeks, approximately 20-50% of patients had missed bladder tumors away from the primary lesion [2,3]. Patients and healthcare providers would benefit from the ability to verify that a diagnostic cystoscopy has completely evaluated their bladder.

Another limitation of the current cystoscopic technique is that no images or videos are routinely saved. This leads to a scenario where the only record of the procedure is the text written by the urologist. It is worth stressing that in the current system a urologist cannot easily compare bladder lesions over time to differentiate the evolution of lesions and better determine which lesions to biopsy. This is important because the false-positive rate for a biopsy of a suspicious mass following a cystoscopy performed for evaluation of microscopic hematuria is approximately 55% [4].

Prior attempts to perform three-dimensional (3D) renderings of the bladder were limited by the technologies available at the time. Lurie et al. utilized structure from motion (SfM), a computational technique that uses a sequence of 2D images to estimate the 3D coordinates of points in a scene, as well as the camera position, to reconstruct a 3D representation. However, they found that the computational time required was too long for routine use and that the model was highly specific to its intended scenes [5]. Soper et al. developed a novel robotic cystoscopic, which was tested on a pig bladder with high fidelity; however, implementation would require costly new devices to be distributed widely [6].

One of the most promising recent innovations in 3D rendering technology is neural radiance field (NeRF) processing, a deep learning model that generates a 3D representation of a scene from a set of 2D images. NeRFs can be applied to a wide variety of scenes, as they are retrained specifically for each individual dataset of images from a scene [7]. The model offers a clear advantage over technologies used in the past, as the datasets it uses do not require any specialized software or hardware for collection, any set of 2D images taken with the same equipment can be used.

In this paper, we hypothesized that using NeRF can successfully perform post-procedural 3D renders of diagnostic cystoscopy footage with sufficient fidelity independent of the type of cystoscope utilized.

This article was previously presented as a meeting abstract at the 2024 American Urological Association (AUA) Annual Meeting on May 3, 2024, and published as an abstract in the* Journal of Urology*.

## Technical report

Patients

With approval from the Institutional Review Board of the University of California, San Diego (approval 806149) and informed consent obtained from all subjects, we recorded two diagnostic cystoscopies in consecutive patients undergoing diagnostic flexible cystoscopy for high-risk asymptomatic microscopic hematuria. Patients were eligible if they were over 18 years old and had no history of urinary tract anomalies or recurrent urinary tract infections. We used standard sterile technique fully described here [8], although we did take extra precaution to pan slowly throughout the bladder to minimize blur artifacts. In the first procedure, we used an Ambu® aScope™ single-use flexible cystoscope and, in the second, a Richard Wolf digital cystoscope. All patients were followed for any post-procedural complications for two weeks.

Image processing and 3D rendering

We converted the videos to images at 30 frames per second and manually curated the images to choose approximately 100 representative images, minimizing motion blur and spanning a large segment of the bladder. COLMAP, a general-purpose, end-to-end image-based 3D reconstruction pipeline, was used to estimate camera position in the video in relation to the anatomy [9]. The images and estimated camera positions were then inputted into NVIDIA Instant Neural Graphics Primitives (iNGP), a NeRF algorithm that uses multiresolution hash encoding with a compact neural network for significantly faster convergence, to reconstruct the bladder and render novel, unseen views to a few seconds. iNGP was trained on the image set. The rendering process was executed utilizing a central processing unit (CPU) featuring the 11th-generation Intel® Core™ i9-11900F, in conjunction with a graphics processing unit (GPU) equipped with the Nvidia GeForce RTX 3090.

Quality metrics

We computed the peak signal-to-noise ratio (PSNR) and structural similarity index (SSIM) to assess the quality and fidelity of the 3D rendering and compared these metrics for the renderings using the different cystoscopes. The PSNR is calculated by taking the negative logarithm of the mean squared error (MSE) between the two images. This metric provides an absolute measure of the image quality, with higher values indicating greater similarity. By contrast, the SSIM is a perceptual metric that takes into account the interdependence between neighboring pixels and ranges in value from -1 to 1, where 1 indicates perfect fidelity [10]. In addition, we tracked approximate computation efficiency for COLMAP, the NeRF model training, and the time required to render novel views for each 3D rendering.

Results

**Figure 1:**
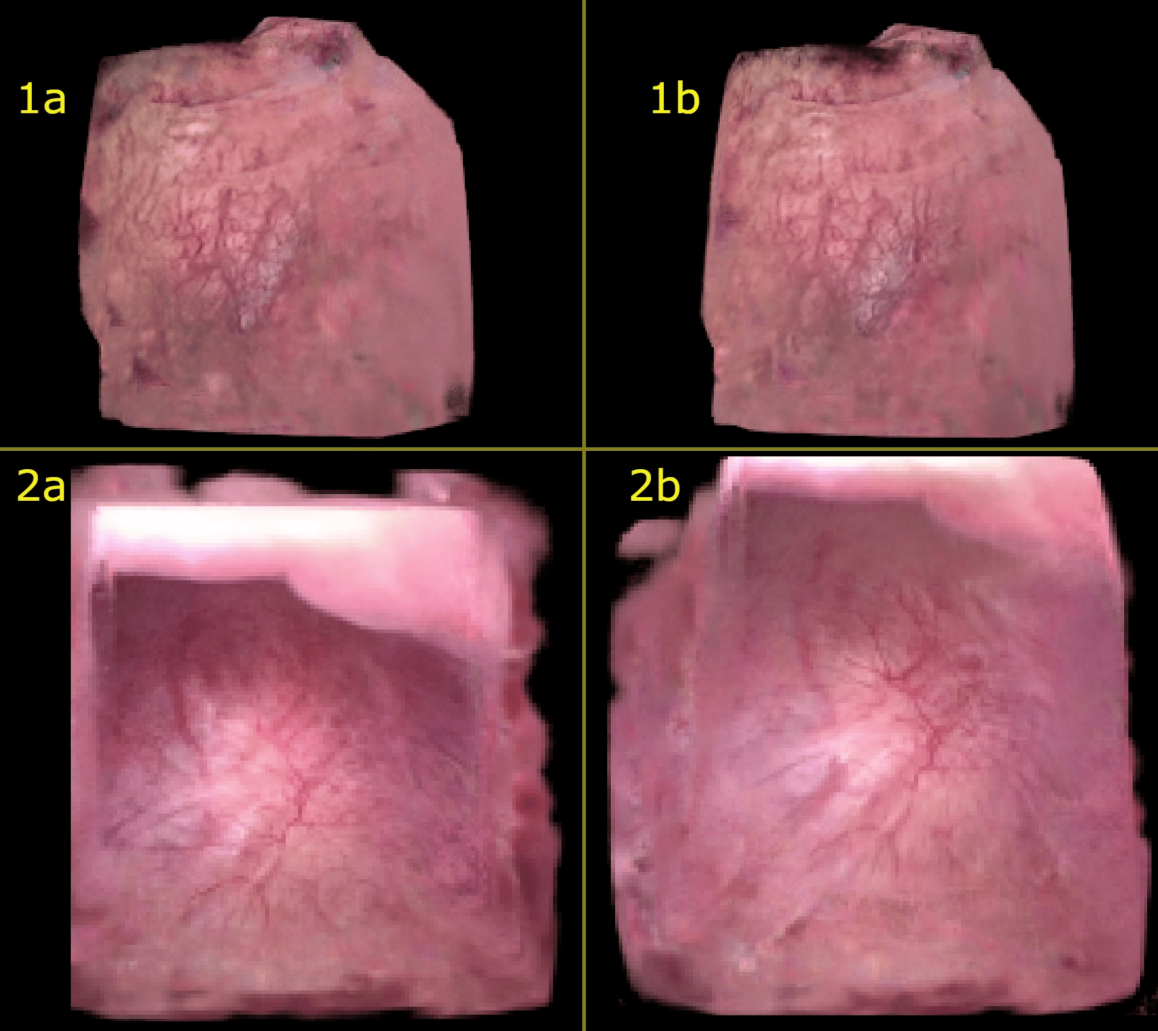
3D rendering static sample images Top row – two views of 3D rendering from Wolf digital cystoscope (1a and 1b). Bottom row – two views of 3D rendering from Ambu disposable scope (2a and 2b).

Both patients underwent successful diagnostic flexible cystoscopies with no abnormalities noted. Neither patient had any complication at two-week follow-up. Both videos were able to be utilized for 3D rendering using iNGP. Fidelity metrics were calculated across approximately 100 rendered views per case, comparing each rendered image to the corresponding frame from the input video. The rendering derived from the Wolf cystoscopy had a PSNR = 29.8 (min = 27.2, max = 32.6) and SSIM = 0.89. Similarly, the rendering derived from the Ambu® cystoscopy had a PSNR = 31.3 (min = 27.1, max = 35.1) and SSIM = 0.90, with the static sample images presented below (Figure 1).

In addition, we exported a video manipulating the 3D rendering from the Ambu cystoscope to demonstrate the novel views achieved with 3D rendering (Video 1).

**Video 1:** Demo rendering video Video footage of a user maneuvering around the 3D bladder rendering to observe novel views.

With respect to computational efficiency, COLMAP requires approximately one minute to ascertain the precise positioning of cameras for a set of 50 to 100 images. The training process associated with the NeRF model generally converges within 10 seconds; however, for the purpose of maintaining standardized conditions, we opted to extend the training duration to one minute. In the context of image rendering using the Wolf cystoscope, we observed an average processing time of 4.89 seconds per image, while the Ambu cystoscope required an average of 8.65 seconds per image (Table 1).

**Table 1:** Summary of results

Cystoscope used	PSNR	SSIM	Average processing time per image (seconds)
Richard-Wolf	29.8 (27.2-32.6)	0.89	4.89
Ambu® aScope	31.3 (27.1-35.1)	0.90	8.65

## Discussion

Cystoscopy remains the gold standard for the evaluation of the bladder. While invasive, it provides excellent visualization of the bladder mucosa, which is crucial for detecting bladder cancer. However, limitations with this technology include an inability to easily compare indeterminate bladder lesions over time and an inability to objectively assess the completeness of the procedure. A rapid 3D rendering that could be reviewed on demand would resolve these limitations. Therefore, we utilized NeRF to attempt to address these shortcomings with no additional expensive instrumentation or cumbersome changes to a urologist workflow. Herein, we found that both a disposable Ambu cystoscope and a reusable digital Richard Wolf cystoscope provided adequate images for successful 3D rendering of a segment of the bladder within a limited timeframe.

The primary challenge faced by previous research teams was reconstructing 3D images of the bladder without real-time tracking of the cystoscope position in reference to the bladder, while adjusting for a dynamic light source. We found that NeRF successfully leveraged the power of neural networks to quickly reconstruct 3D bladder segments. However, in each rendering, as we attempted to increase the area of the bladder included, the rendering failed to find enough overlap in the images to generate a rendering. This may be due to both the fact that COLMAP provides imperfect estimates of camera position, and also that NeRF excels when multiple viewpoints of a single region is available, whereas the current environment provides several viewpoints of a large region. This challenge is not unique to NeRF and is a basic challenge for all 3D scene reconstruction techniques. Recent advances in NeRF with sparse viewpoints and noisy camera poses can be adapted to this problem to improve performance in the future [11].

In addition, while there is no universally accepted PSNR or SSIM threshold denoting clinical utility for cystoscopic bladder renderings, the radiology and medical imaging literature frequently reports PSNR values around 35 dB for compression-related tasks, although these values are modality-specific and vary between MRI, CT, and X-ray imaging [12]. In our study, PSNR values of ~30-31 dB and SSIM values of ~0.89-0.90 likely fall within the lower end of these commonly referenced ranges. However, given the limited sample size (n = 2), these findings should be interpreted as a proof-of-concept rather than a statistical comparison, and further validation will be necessary to determine clinically meaningful PSNR and SSIM thresholds in the context of 3D reconstruction from cystoscopic video footage.

Technical modifications whereby the urologist pauses in 75-100 locations may help improve the rendering but would be cumbersome and frustrating for patients and providers alike. In addition, to effectively determine if the entire bladder had been surveyed, the process of image curation would need to be automated, which would require substantially more patient data. Nevertheless, we feel that with further development of this technology can be utilized to create complete 3D renderings of the bladder and eventually to assess the completeness of the performed procedure. Together these features will enable both a more objective assessment of the completeness of an individual cystoscopy procedure, as well as provide an easy to analyze artifact of the cystoscopic exam which could be used to compare bladder lesions over time.

## Conclusions

Our study found that 3D renderings from cystoscopy videos can achieve reasonable fidelity independent of the equipment used. However, major limitations persist. The most important among them is the need for manual curation of representative, high-quality images form the source cystoscopy videos, a process that introduces potential bias and limits reproducibility. In addition, the rendered reconstructions often represent relatively small segments of the bladder. Despite these challenges, we believe that with further refinement, this technology has the potential to scale effectively, creating 3D renderings of cystoscopies that enable comprehensive evaluations of completeness and quality. Furthermore, it could significantly enhance the ability to compare cystoscopies performed on the same patient over time, offering valuable insights into disease progression and treatment efficacy.
